# Primary renal lymphoma: unique presentation in a rare disease

**DOI:** 10.1093/jscr/rjab011

**Published:** 2021-03-08

**Authors:** Mohamed Youssef, Faustina Davis, Praveen Rao

**Affiliations:** Department of Urology, Blackpool Victoria Hospital, Blackpool, UK; Department of Urology, Blackpool Victoria Hospital, Blackpool, UK; Department of Urology, Blackpool Victoria Hospital, Blackpool, UK

## Abstract

As the kidney is an extra-nodal organ and does not have lymphatic tissues, the existence of primary renal non-Hodgkin’s lymphoma has been continuously questioned. It is very rare. However, differentiation between renal cell carcinoma and renal lymphoma is necessary in patients presenting with solitary renal mass. We present a 70-year-old-man who presented with a renal mass and was diagnosed with diffuse large B-cell lymphoma. We feel that this case report may be of benefit to clinicians who may encounter a similar scenario.

## INTRODUCTION

Diffuse large B-cell lymphoma (DLBCL) is the most common subtype of non-Hodgkin’s lymphoma (NHL), and constitutes 25% of all NHL cases. The disease usually presents with a rapidly enlarging symptomatic mass, and the diagnosis is made on an excision biopsy. It is a morphological and immune-phenotypical diagnosis [1].

Renal NHL is most commonly a part of a disseminated disease, and primary renal lymphoma (PRL) is thought to be a rare disease, with no >70 cases reported in the literature. Clinically and radiologically, PRL may be confused with renal cell carcinoma (RCC) [2].

The diagnosis of PRL should be confirmed by the presence of a renal mass, with no evidence of extra lymphomatous involvement in the absence of a leukemic blood picture. Kidney biopsy is the gold standard for the diagnosis [3]. Hypercalcaemia is considered to be rare in B-cell non-Hodgkin’s lymphoma (B-NHL) [4].

Current treatment—as any other DLBCL—typically includes R-CHOP, which consists of the traditional CHOP (Cyclophosphamide, doxorubicin, vincristine and Prednisone), to which rituximab has been added [5].

## CASE REPORT

A 70-year-old man presented to his doctor in the community, feeling generally unwell, with loss of appetite and lethargy for 3 weeks. It was associated with intermittent left sided abdominal pain and his wife noticed that he was occasionally confused. He had intentionally lost weight, but had no fever or night sweats. He was an ex-smoker of 40 years, with hypertension, but otherwise fit and lives a healthy life. Routine blood tests were done in the community; they came back showing he had raised corrected calcium of 3.13 mmol/L (normal range 2.20–2.60). He was immediately sent to the local hospital for further investigation and management of his hypercalcaemia. PTH was normal, and Bence Jones protein negative. He was managed with intravenous fluids and his Bendroflumethiazide was stopped. At the time, it was thought that his hypercalcaemia was most likely secondary to his diuretic. His hypercalcaemia improved and he was discharged home with a computed tomography (CT) scan of his chest, abdomen and pelvis ordered as urgent outpatient to rule out malignancy.

**Figure 1:**
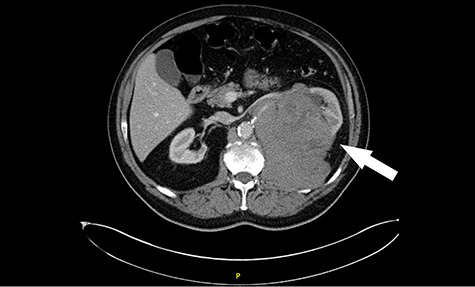
Computed tomography (CT) revealing a large insinuating left renal soft tissue mass measuring 140 × 105 mm.

**Figure 2:**
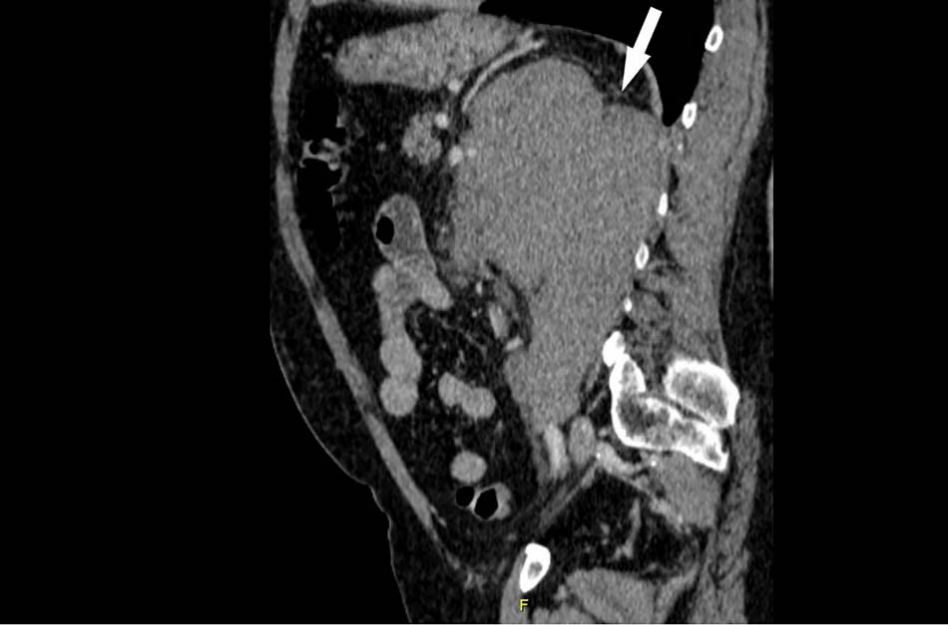
Computed tomography (CT) sagittal section showing predominant bulk of the mass is extrarenal that posteriorly breaches the fascia to involve the diaphragm and psoas muscle. Medially it reaches the intervertebral foramen.

His CT scan identified large left renal tumour invading psoas muscle. Left mid ureteric tumour as well. Multiple regional nodes. No hepatic, pulmonary or osseous metastasis. The scan was highly suggestive of metastatic transitional cell carcinoma (Figs. 1 and 2).

While awaiting further investigation, he presented to the local hospital again with severe left flank pain and difficulty passing urine. He underwent an urgent ureteroscopy for insertion of a retrograde ureteric JJ stent in his left side and biopsy for histological diagnosis. His ongoing hypercalcaemia was managed with intravenous fluids and bisphosphonate. Histology confirmed diffuse large B cell lymphoma (DLBCL).Immunohistochemical analysis revealed BCL-2 + BCL-6 +/-CD10 -CD20 + CD23—CD30—CD5—CD79 + CyclinD1—IRF4 + Ki67 70% LMP-1—Post Germinal Centre B-cell Phenotype—DLBCL.

We have planned to give him six cycles of R-CHOP chemotherapy. Renal disease is a high risk site for CNS relapse of lymphoma, so we have undertaken a diagnostic lumbar puncture. His cytospin was clear. Despite this we do plan to give him high-dose methotrexate to reduce risk of CNS relapse.

So far, he is tolerating therapy well so far and we plan to deliver the next rounds of chemotherapy as an outpatient. We will do a CT scan after six cycles, or earlier if clinically indicated.

## DISCUSSION

PRL is a rare disease. It is defined as lymphoma arising in the renal parenchyma and not resulting from the invasion of an adjacent lymphomatous mass [6].

Fever in younger patients is the main symptom (56%). Patients between 18 and 50 years old commonly present with abdominal and flank pain (62%). Weight loss and gross haematuria are the most common symptoms (37%) if the patient is older than 50. The highest survival rate (mean, 62.8 months) is seen in patients aged between 18 and 50 compared with patients aged from 0 to18 years old (mean, 17.6 months) and >50 years (mean, 48.2 months) [7].

Hypercalcaemia is supposed to be rare in B-NHL but several individual case reports have appeared over the years [9, 10]. No systematic study of hypercalcaemia in B-NHL has been published. Incidence of 7% and 8.5% of hypercalcaemia have been reported in newly diagnosed patients with high grade B-NHL [11].

The prognosis is reported poor universally. Median survival is less than a year. PRL is considered as a systemic disease, presenting with renal manifestation. However, nephrectomy can be avoided if a preoperative diagnosis is made. In patients with atypical features of RCC, it is recommended that a preoperative percutaneous renal biopsy is undertaken. The sensitivity and specificity of renal biopsy are fairly high. The treatment of renal lymphoma depends on the primary histological subtype. The addition of Rituximab to the standard CHOP chemotherapy may improve the dismal outcome reported sofar.

Our patient presented to his GP feeling unwell, blood tests showed hypercalcaemia. Further imaging showed solitary large mass in the left kidney. Our initial impression was that the mass in the kidney represented primary RCC or TCC. The ureteric biopsies however confirmed diagnosis of PRL (substantiated by immunohistochemistry).

This case highlights the importance of making the differentiation between PRL and RCC which can have similar clinical and radiological features.

Although rare, recognizing the entity of PRL could avoid unnecessary nephrectomies.
